# ANU-ADRI scores, tau pathology, and cognition in non-demented adults: the CABLE study

**DOI:** 10.1186/s13195-024-01427-6

**Published:** 2024-03-26

**Authors:** Shan Yin, Pei-Yang Gao, Ya-Nan Ou, Yan Fu, Ying Liu, Zuo-Teng Wang, Bao-Lin Han, Lan Tan

**Affiliations:** Department of Neurology, Qingdao Municipal Hospital, Qingdao University, No.5 Donghai Middle Road, Qingdao, China

**Keywords:** Alzheimer’s disease, ANU-ADRI, Cerebrospinal fluid, Biomarkers

## Abstract

**Background:**

It has been reported that the risk of Alzheimer’s disease (AD) could be predicted by the Australian National University Alzheimer Disease Risk Index (ANU-ADRI) scores. However, among non-demented Chinese adults, the correlations of ANU-ADRI scores with cerebrospinal fluid (CSF) core biomarkers and cognition remain unclear.

**Methods:**

Individuals from the Chinese Alzheimer’s Biomarker and LifestyLE (CABLE) study were grouped into three groups (low/intermediate/high risk groups) based on their ANU-ADRI scores. The multiple linear regression models were conducted to investigate the correlations of ANU-ADRI scores with several biomarkers of AD pathology. Mediation model and structural equation model (SEM) were conducted to investigate the mediators of the correlation between ANU-ADRI scores and cognition.

**Results:**

A total of 1078 non-demented elders were included in our study, with a mean age of 62.58 (standard deviation [SD] 10.06) years as well as a female proportion of 44.16% (n = 476). ANU-ADRI scores were found to be significantly related with MMSE (β = -0.264, *P* < 0.001) and MoCA (β = -0.393, *P* < 0.001), as well as CSF t-tau (β = 0.236, *P* < 0.001), p-tau (β = 0.183, *P* < 0.001), and t-tau/Aβ42 (β = 0.094, *P* = 0.005). Mediation analyses indicated that the relationships of ANU-ADRI scores with cognitive scores were mediated by CSF t-tau or p-tau (mediating proportions ranging from 4.45% to 10.50%). SEM did not reveal that ANU-ADRI scores affected cognition by tau-related pathology and level of CSF soluble triggering receptor expressed on myeloid cells 2 (sTREM2).

**Conclusion:**

ANU-ADRI scores were associated with cognition and tau pathology. We also revealed a potential pathological mechanism underlying the impact of ANU-ADRI scores on cognition.

**Supplementary Information:**

The online version contains supplementary material available at 10.1186/s13195-024-01427-6.

## Introduction

The most common type of dementia is the Alzheimer’s disease (AD) [1], which is neuropathologically characterized by accumulated amyloid-β (Aβ) peptide protein and intraneuronal tangles of hyperphosphorylated micro tubule-associated protein tau [2]. The number of dementia patients has been estimated to be 152 million by 2050 worldwide, with an annual global cost of around US$1 trillion annually [3]. However, due to the lack of effective treatments for AD, validated risk assessment and prevention remain earlier and more desirable strategies [4]. Published studies provided possibility that imminent dementia in a high-risk individual could averted by healthy lifestyle and other environmental adjustments [2, 5]. Further evidences from randomized controlled trials (RCT) showed that multidomain interventions exerted optimal preventive effects on for cognitive decline [6, 7]. Although several lifestyle pattern and AD risk assessment instruments were developed for predicting AD [8–10], the most valuable assessment tool was uncertain.

The Australian National University Alzheimer Disease Risk Index (ANU-ADRI) was a self-report instrument for assessing AD risk, which encompasses 4 protective and 11 risk factors for AD (e.g. age, gender, year of education, smoking, diabetes, etc.) [11–13]. The previous study demonstrated that ANU-ADRI scores could predict AD and dementia in three cohorts [14]. Further evidences suggested ANU-ADRI was a better instrument for predicting dementia than Cardiovascular Risk Factors, Aging and Dementia study (CAIDE) and the Study on Aging, Cognition and Dementia (AgeCoDe) model [15, 16]. However, the mechanism underlying the relationship between ANU-ADRI and AD pathology remains unclear. Microglia-related neuroinflammation might explain how some risk factors in ANU-ADRI scores (e.g., depression and smoking) influence AD pathology [17, 18]. Triggering receptor expressed on myeloid cell 2 (TREM2) is expressed mainly by microglia cell in the central nervous system (CNS) [19]. The soluble TREM2 (sTREM2) could be detected in cerebrospinal fluid (CSF), which is shed from DAM following the cleavage of TREM2. And CSF sTREM2 was regarded as an underlying and novel marker of neuroinflammation in AD [20].

Therefore, our study aimed: (1) to assess the associations of ANU-ADRI scores with CSF core biomarkers and cognition; (2) to evaluate whether the relationship of ANU-ADRI scores with cognition was mediated by CSF AD biomarkers; (3) to investigate whether the relationship of ANU-ADRI scores with cognition was mediated by CSF core biomarkers and sTREM2.

## Method

### The CABLE study and participants

The Chinese Alzheimer’s Biomarker and LifestyLE (CABLE) study is a large-scale ongoing and independent cohort study, which mainly focused on genetic and environmental risk factors as well as biomarkers of AD in northern Han Chinese population. All individuals in the CABLE study were aged 40 to 90 years old and were recruited from Qingdao Municipal Hospital, Shandong Province, China. Individuals will be excluded if the following exclusion criteria are met: (a) central nervous system infection, epilepsy, multiple sclerosis, or other major neurological disorders; (b) major psychological disorders; (c) severe systemic diseases (e.g., malignant tumors); (d) family history of genetic diseases. The CABLE study was granted approval by the Ethic Committee of Qingdao Municipal Hospital and was undertaken in line with the Declaration of Helsinki. All participants provided written consent.

Participants from CABLE study had data on CSF AD biomarkers and risk and protective factors in ANU-ADRI scores. A total of 2,466 individuals from the CABLE were evaluated. 723 participants who did not have available CSF AD biomarkers were excluded and 665 participants were excluded without data on factors in ANU-ADRI (e.g. education, alcohol, depression, smoking, etc.). So far, dementia cases have not been included in the study. Finally, our study included 1,078 non-demented (including cognitively normal [CN], subjective cognitive declined [SCD], and mild cognitive impairment [MCI]) elders from the CABLE study. The detailed diagnosis criteria are reported in supplementary Table 6. All the individuals included in this study underwent comprehensive clinical, neuropsychological, psychosocial, and psychiatric examinations as well as collection of biological specimens (including blood and CSF specimens).

### Measurements of ANU-ADRI metrics

The ANU-ADRI is a novel self-report AD risk assessment tool developed based on an Evidence-Based Medicine approach, which includes 15 risk and protective factors for AD [11]. Age, gender educational level, smoking, and drinking habits, fish intake and exercise frequency were self-reported, with the electronic medical record system as a reference. According to the years of education, educational status was defined as low (< 8 years), medium (8–11 years), as well as high (> 11 years). As for smoking habits, participants were divided into never smoking, ever smokers, and current smokers. Alcohol status was defined by alcohol intake, with response options of no-drinkers and drinkers [13]. According to exercise frequency, physical activity status was defined low (never or occasionally), medium (once a week or several times a week), as well as high (daily frequent). The frequency of fish intake was defined as “Never”, “Some days” (once a week or occasionally), “Most days” (several times a week), and “Every day”. We evaluated the depression symptoms of participants based on the Hamilton Depression Scale (HAMD; depression: HAMD ≥ 7). BMI and cholesterol would not be included in the ANU-ADRI scores for participants over 60 years old. The body mass index (BMI) was calculated as weight divided by height in meters squared. After fasting for at least 8 h, the plasma total cholesterol was tested by an experienced doctor using the enzymatic method at the laboratory of the Department of Clinical Chemistry at Qingdao Municipal Hospital in China.

All ANU-ADRI metrics could be operationalized in the CABLE study except for the social engagement, cognitive activity, and occupational pesticide exposure. A subset of ANU-ADRI factors could still predict the development of dementia [21]. The score of each item was computed by the standardized beta-weights based on the odds risks of pooled effect sizes from published meta-analyses [11]. The composite ANU-ADRI scores, which were calculated from the sum of 11 components, were classified as low (< X－1 standard deviation [SD]), intermediate (between X ± 1 SD), and high risk (> X + 1 SD). The all metrics of ANU-ADRI, assigned weights, and detailed operationalization in CABLE dataset were shown in Table 1.

**Table 1:** Full details of the ANU-ADRI score mapping in the CABLE Study

Goal/Metric	Points	
Age	Male	Female
65 years	0	0
65—70 years	1	5
70—75 years	12	14
75—80 years	18	21
80—85 years	26	29
85—90 years	33	35
≥ 90 years	38	41
Education		
> 11 years	0	
8—11 years	3	
< 8 years	6	
BMI (aged < 60)		
< 25	0	
25- < 30	2	
≥ 30	5	
Diabetes		
No	0	
Yes	3	
Symptoms of depression		
HAMD ≤ 7	0	
HAMD > 7	2	
Total cholesterol		
< 6.2mmol/L	0	
≥ 6.2mmol/L	3	
History of a traumatic brain injury		
No	0	
Yes	4	
Smoking		
Never	0	
Ever smoking	1	
Current	4	
Alcohol intake		
Non-drinker	0	
Drinker	-3	
Physical activity		
Low: never or occasionally	0	
Medium: once a week or several times a week	-2	
High: every day	-3	
Fish intake		
Never	0	
Some days	-3	
Most days	-4	
Every day	-5	

*BMI* Body mass index, *HAMD* Hamilton Depression Scale

### APOE and cognitive assessments

DNA was extracted from fasting blood specimens using the QIAamp® DNA Blood Mini Kit (250). The restriction fragment length polymorphism technique was used for genotyping by selecting specific loci (*rs7412* and *rs429358*) linked with *APOE ε4* status. Global cognition of participants was assessed by the Chinese version of Mini-Mental State Examination (CM-MMSE) and the Montreal Cognitive Assessment (MoCA).

### CSF AD biomarkers and sTREM2

The CSF specimens were collected via lumbar puncture in 10 mL polypropylene tubes, and then were delivered to the lab within two hours. CSF samples were centrifuged at 2000 × g for 10 min and stored in refrigerator at -80 °C. The thaw/freezing cycle was required less than two times. CSF β-amyloid42 (Aβ42, catalog number: 81583), total tau (t-tau, catalog number: 81579), as well as phosphorylated tau (p-tau, catalog number: 81581) were measured with the enzyme-linked immunosorbent assay (ELISA) kits (INNOTEST; FUJIREBIO, Ghent, Belgium) and CSF sTREM2 was measured using the ELISA kits (Human TREM2 SimpleStep ELISA kit; Abcam, NO. Ab224881). Each measurement was conducted by specialist technicians who were blinded to clinical information. The within-batch coefficient of variation (CV) was < 5% (mean CV 4.55% for Aβ42, 4.45% for tau, and 3.18% for p-tau).

### Statistical analyses

In this study, all continuous variables were normalized using the Box–Cox transformations and were standardized using Z-score. We further excluded the extreme values of the CSF biomarkers (outside of 3 SDs).

The chi-square test and the one-way analysis of variance (ANOVA) were applied to compare baseline demographical characteristics, *APOE ε4* status, cognition, CSF AD biomarkers, and inflammatory markers between the three risk groups. We further explored the differences in CSF AD biomarkers and cognition between three risk groups using one-way ANOVA and Tukey Honest significant post hoc analyses of variance. Moreover, multiple linear regression (MLR) models were performed to test the relationships of the ANU-ADRI scores with CSF AD biomarkers (Aβ42, t-tau, p-tau_,_ t-tau/Aβ42, p-tau/Aβ42) and cognition (MMSE and MoCA scores) after adjustment for *APOE ɛ4* allele carrier statuses. Sensitivity analysis was conducted by additionally adjusting for three covariates, including stroke, hypertension, and comorbidities of coronary heart disease. The interaction analyses were applied to examine whether the associations between ANU-ADRI scores and cognition were affected by *APOE ε4* status.

Furthermore, in order to test whether CSF AD biomarkers could mediate the association between ANU-ADRI scores (the independent variable) and cognition (dependent variable), three regression equations should be evaluated: (a) regressing CSF AD biomarkers on the ANU-ADRI scores; (b) regressing cognition (MMSE and MoCA) the ANU-ADRI scores; (c) regressing cognition on the ANU-ADRI scores and CSF AD biomarkers [22]. In order to establish the mediation models that used CSF AD biomarkers as mediators, the following criteria must be met: (1) ANU-ADRI scores were significantly corelated with CSF core biomarkers; (2) ANU-ADRI scores were significantly linked with cognitive score; (3) Several biomarkers of AD pathology were significantly related with cognitive score; and (4) the relationships of ANU-ADRI scores with cognitive score were attenuated when CSF AD biomarkers were added in the regression model. The indirect effects or attenuation were evaluated. In each mediation model, each path was controlled for the same covariate (*APOE* status). Since a previous study has suggested that tau pathology is linked to an elevated CSF sTREM2 levels [23]. Thus three mediation models with structural equation model (SEM) were applied in our study. The serial multiple mediation analysis was conducted to investigate whether the correlations of ANU-ADRI scores with cognitive score were mediated by CSF p-tau/t-tau and CSF sTREM2 after adjusting *APOE* status. Two other mediation analyses were used to examine whether the associations of ANU-ADRI scores with cognitive score were mediated only by CSF sTREM2 or CSF p-tau/t-tau after adjusting *APOE* status. For each mediation model, ANU-ADRI scores were regarded as the independent variable and cognition (including MMSE and MoCA scores) was regarded as the dependent variable.

A two-sided *P* value < 0.05 was considered as statistical significance. The “car”, “stats”, “mediation”, “lavaan”, and “ggplot2” packages in R (version 4.2.1) software were applied to conduct the statistical analyses and graph generation in this study.

## Results

### Characteristics of participant in the CABLE study

A total of 1,078 individuals from the CABLE study were included in the cross-sectional study. Demographical and clinical characteristics of our participants were described in Table 2. The total individuals had a mean age of 62.58 years [SD 10.06], a female proportion of 44.16%, as well as an *APOE ε4* carrier proportion of 15.90%. The mean ANU-ADRI score was 4.65[SD 9.00]. According the total ANU-ADRI scores, they were grouped into low risk group (n = 110), intermediate risk group (n = 793), and high risk group (n = 175). Compared with low and intermedium risk groups, participants from the high risk group tended to be older and less-educated.

**Table 2:** Characteristics of participants across ANU-ADRI categories

Characteristics	Total	ANU-ADRI categories			*P* value
		Low risk	Intermediate risk	High risk	
N	1078	110	793	175	—
Age (years), mean (SD)	62.58 (10.06)	57.89 (7.27)	60.19 (8.57)	76.35 (5.25)	** < 0.001**^a^
Gender, female (%)	476 (44.16%)	20 (18.20)	377 (47.50)	79 (45.10)	** < 0.001**^b^
Education (years), mean (SD)	9.30 (4.36)	13.42 (2.32)	9.07 (4.19)	7.77 (4.61)	** < 0.001**^a^
*APOE* ε4, yes (%)	149 (15.90%)	17 (18.70)	110 (15.90)	22 (14.40)	0.674^b^
CM-MMSE, mean (SD)	27.07 (3.18)	28.64 (1.63)	27.19 (3.04)	25.54 (3.86)	** < 0.001**^a^
MoCA, mean (SD)	22.39 (4.76)	22.37 (4.76)	22.39 (4.75)	22.38 (4.76)	** < 0.001**^a^
ANU-ADRI score, mean (SD)	4.65 (9.00)	-6.73 (1.46)	2.61 (4.47)	21.04 (6.13)	** < 0.001**^a^
**CSF AD biomarkers**					
Aβ42, mean (SD)	345.48 (214.07)	343.43 (198.54)	345.79 (219.15)	345.39 (201.06)	0.994^a^
P-TAU181, mean (SD)	44.25 (13.67)	41.13 (13.01)	43.58 (13.09)	49.43 (15.44)	**< 0.001**^a^
T-TAU, mean (SD)	197.41 (84.98)	173.61 (75.71)	191.19 (79.41)	241.79 (100.07)	**< 0.001**^a^
P-TAU181/Aβ42, mean (SD)	0.18 (0.13)	0.16 (0.11)	0.18 (0.13)	0.20 (0.15)	**0.043**^a^
T-TAU/Aβ42, mean (SD)	0.80 (0.64)	0.71 (0.61)	0.78 (0.61)	0.98 (0.78)	** < 0.001**^a^
**Central inflammatory markers**					
sTREM2	17,886.95 (7002.11)	17,386.67 (6135.75)	17,402.75 (6922.29)	20,421.25 (7391.03)	** < 0.001**^a^

The statistically significant results have been bolded

*Abbreviations*: *ANU-ADRI* Australian National University-Alzheimer Disease Risk Index, *APOE* ε4 apolipoprotein E genotype ε4, *CM-MMSE* Chinese version of Mini-Mental State Examination, *MoCA* Montreal Cognitive Assessment Scale, *CSF* Cerebrospinal fluid, *AD* Alzheimer’s disease, *Aβ* Amyloid-β, *P-TAU181* phosphorylated tau181, *T-TAU* total tau, *sTREM2* soluble triggering receptor expressed on myeloid cells 2

^a^The difference among the groups was examined by the analysis of variance

^b^The difference among the groups was examined by the chi-square test

### Intergroup differences in CSF AD biomarkers and cognition

**Fig. 1:**
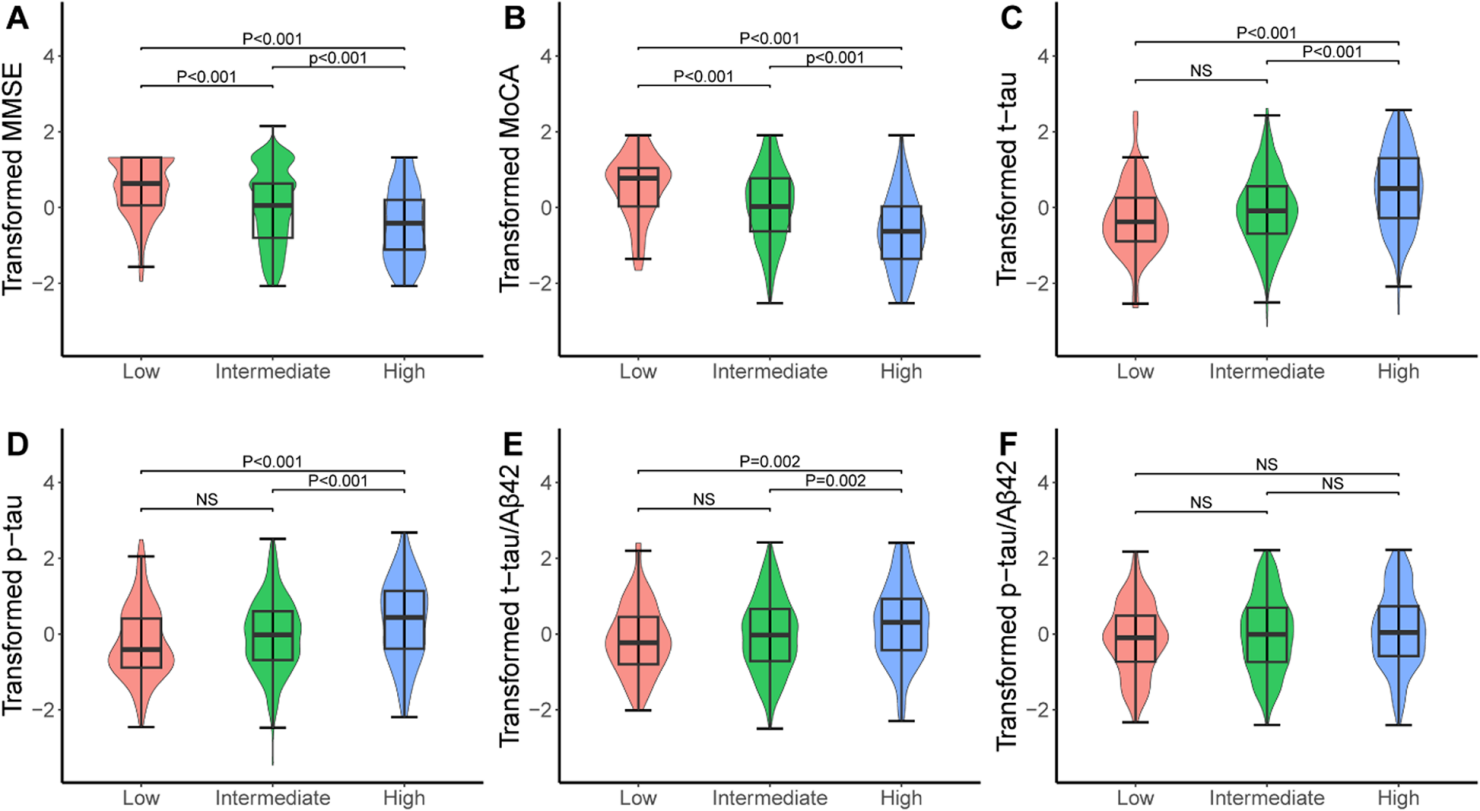
Differences in CSF biomarkers and cognition between the three ANU-ADRI categories. Differences in cognition (**A-B**) and CSF tau-related biomarkers (**C-F**) were examined by the analysis of variance. ANU-ADRI, Australian National University Alzheimer Disease Risk Index; CSF, cerebrospinal fluid; Aβ, amyloid beta; P-tau, phosphorylated tau; T-tau, total tau; MMSE, Mini-Mental State Examination; MoCA, Montreal Cognitive Assessment

The significant differences were found in cognition (both MMSE and MoCA scores) among the three ANU-ADRI risk groups. To be specific, high risk group had worse cognitive performance compared to the low risk group (MMSE: *P* < 0.001; MoCA: *P* < 0.001) and intermedium (MMSE: *P* < 0.001; MoCA: *P* < 0.001) risk group (Fig. 1A-B). As for CSF AD biomarkers, the high risk group had higher levels of CSF AD biomarkers level compared with the low (t-tau: *P* < 0.001; p-tau: *P* < 0.001; t-tau/Aβ42: *P* = 0.002) and intermedium (t-tau: *P* < 0.001; p-tau: *P* < 0.001; t-tau/Aβ42: *P* = 0.002) risk groups (Fig. 1C-E). However, no significant intergroup differences in CSF Aβ42 and p-tau/Aβ42 ratio were found (Supplementary Fig. 1 and Fig. 1).

### Association of ANU-ADRI scores with cognition and several biomarkers of AD pathology

**Fig. 2:**
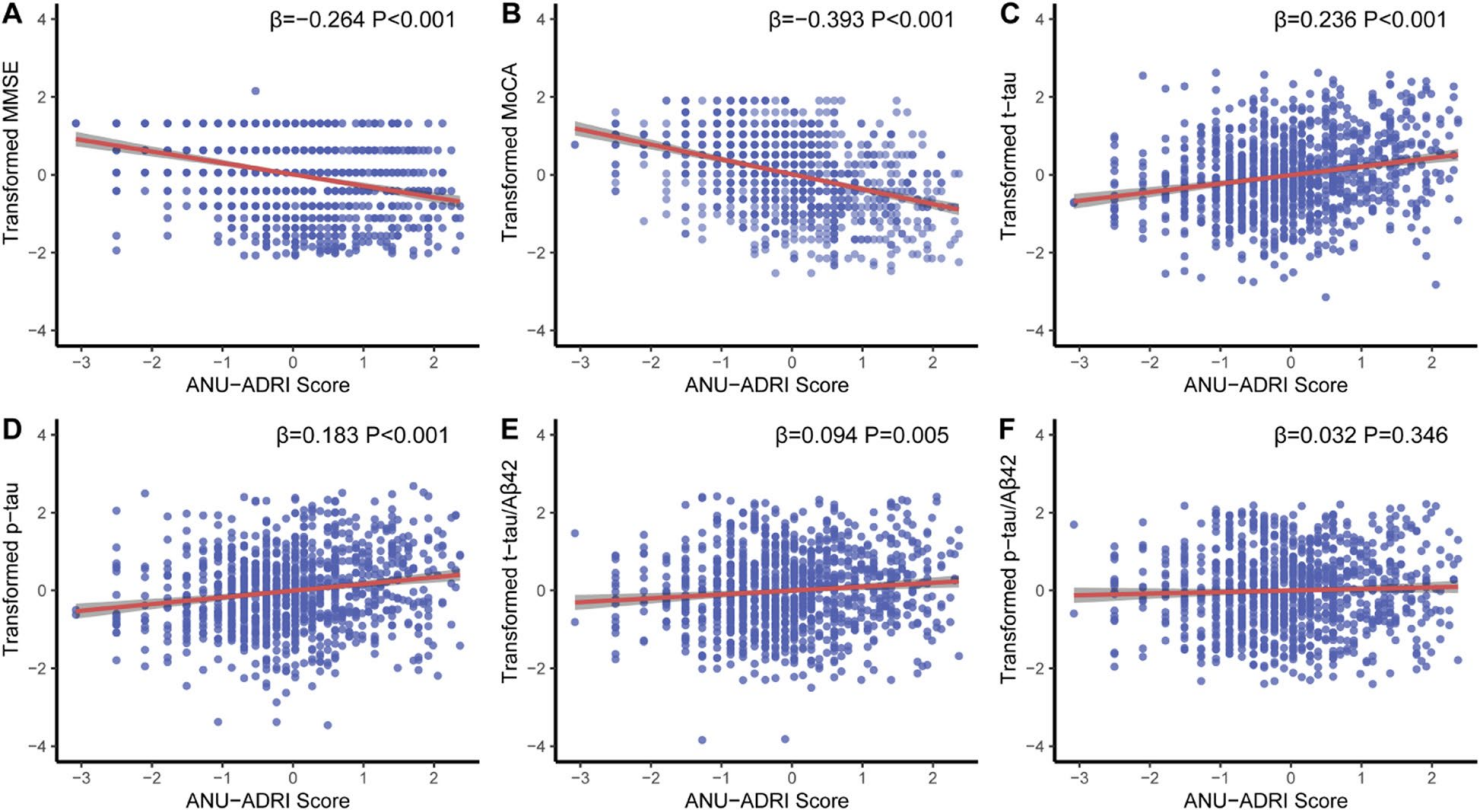
Associations between ANU-ADRI scores and CSF tau-related biomarkers and cognition. Multiple linear regression models were used to examine the associations between the ANU-ADRI scores and cognition (**A-B**) and CSF tau-related biomarkers (**C-F**) adjusting for *APOE ɛ4* allele statuses. ANU-ADRI, Australian National University Alzheimer Disease Risk Index; CSF, cerebrospinal fluid; Aβ, amyloid beta; P-tau, phosphorylated tau; T-tau, total tau; MMSE, Mini-Mental State Examination; MoCA, Montreal Cognitive Assessment

The MLR models were conducted to evaluate the relationships of ANU-ADRI scores with cognitive scores and several biomarkers of AD pathology (Fig. 2). After adjusting for *APOE ɛ4*, significant negative correlations of ANU-ADRI scores with MMSE (*β* = -0.264, *P* < 0.001, Fig. 2A) and MoCA (*β* = -0.393, *P* < 0.001, Fig. 2B) scores were observed. Higher ANU-ADRI scores were associated with increased CSF t-tau (*β* = 0.236, *P* < 0.001, Fig. 2C), p-tau (*β* = 0.183, *P* < 0.001, Fig. 2D), and t-tau/Aβ42 ratio (*β* = 0.094, *P* = 0.005, Fig. 2E). However, ANU-ADRI scores were not associated with Aβ42 (Supplementary Table 1). Sensitivity analysis was conducted by additionally adjusting for the comorbidities of coronary heart disease, stroke, as well as hypertension, which yielded similar findings (Supplementary Table 2). The interaction analyses demonstrated that associations between ANU-ADRI scores and cognition were not affected by *APOE ε4* status (Supplementary Table 3).

### CSF AD biomarkers mediates the association of ANU-ADRI scores with cognition

**Fig. 3:**
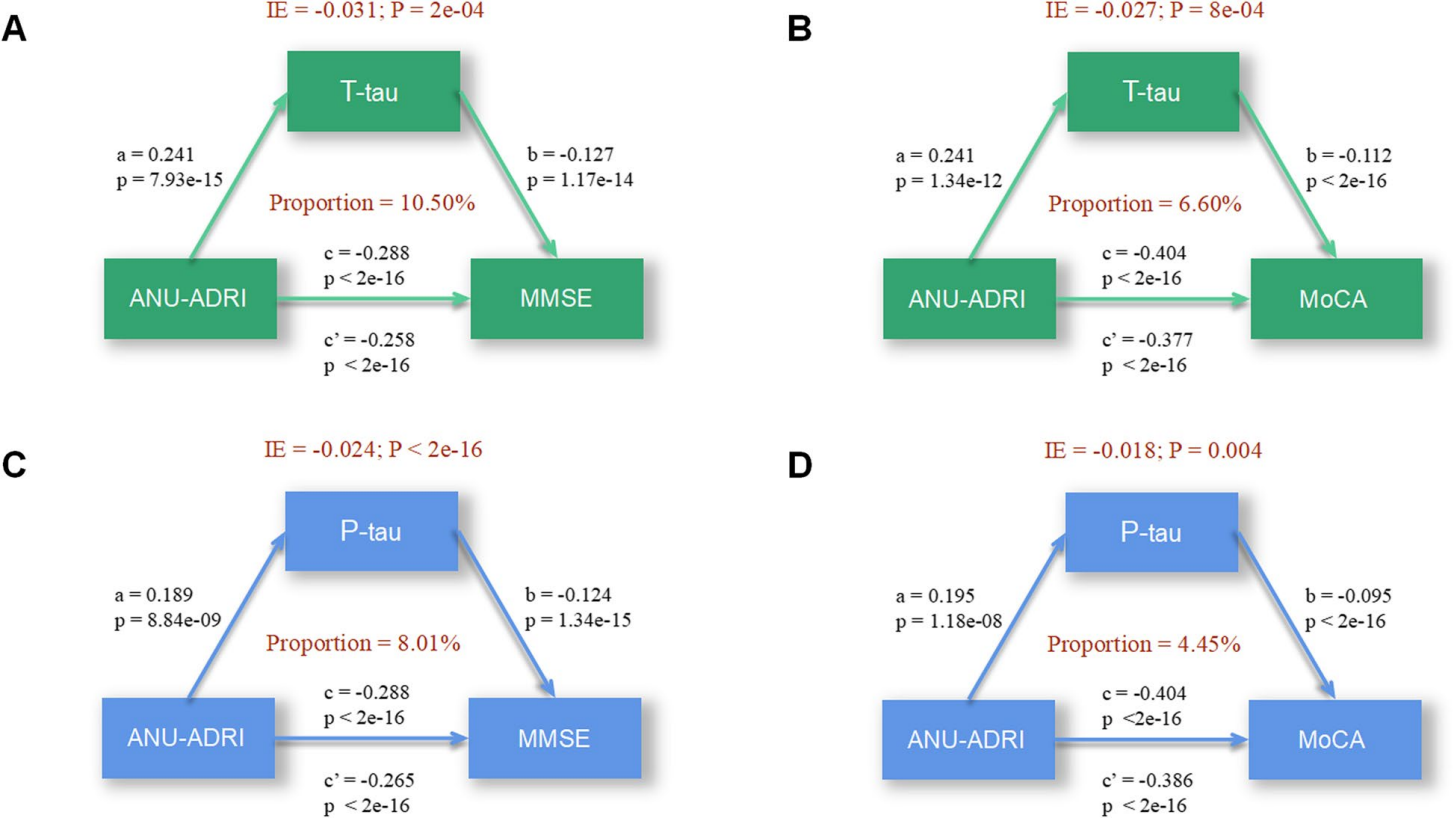
CSF p-tau and t-tau mediated association between ANU-ADRI scores and cognition. Models of mediation for ANU-ADRI scores, cognition (MMSE and MoCA), and CSF biomarkers (p-tau and t-tau), with ANU-ADRI scores as independent variable and CSF biomarkers as mediator and cognition as dependent variable. Each model path was adjusted for *APOE ε4* status. *a* is the effect of the independent variable on mediators; *b* is the effect of mediators on dependent variables after controlling the influence of independent variables; *c* is the total effect of independent variables on dependent variables; *c*’ is the direct effect; IE is the indirect effect. ANU-ADRI, Australian National University Alzheimer Disease Risk Index; CSF, cerebrospinal fluid; P-tau, phosphorylated tau; T-tau, total tau; MMSE, Mini-Mental State Examination; MoCA, Montreal Cognitive Assessment

Our mediation analyses revealed that the correlation of ANU-ADRI scores with MMSE and MoCA scores were partially mediated by CSF t-tau (MMSE score: mediation proportion = 10.50%; MoCA score: mediation proportion = 6.60%, Fig. 3A-B). And p-tau also mediated the relationship of ANU-ADRI scores with cognition (MMSE score: mediation proportion = 8.01%; MoCA score: mediation proportion = 4.45%, Fig. 3C-D).

### Serial mediation between ANU-ADRI scores and cognition

**Fig. 4:**
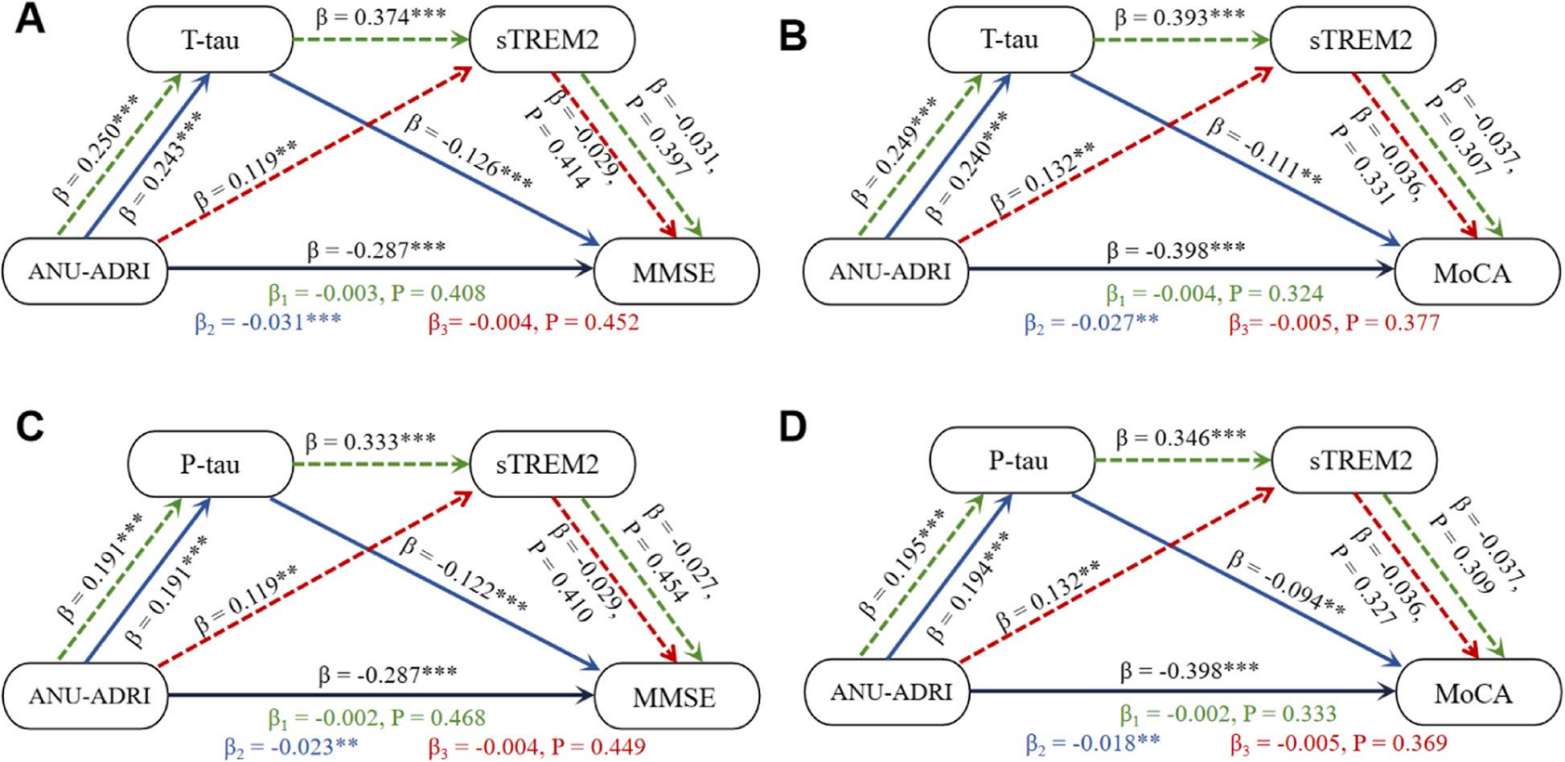
Mediation analysis. **A** Three mediation pathways were conducted between ANU-ADRI scores and MMSE: (1) ANU-ADRI → t-tau → sTREM2 → MMSE; (2) ANU-ADRI → t-tau → MMSE; (3) ANU-ADRI → sTREM2 → MMSE. The serial mediation pathway via t-tau and sTREM2 was not significant (*β*_*1*_ = -0.003, *P* = 0.408). sTREM2 was not significant mediator for this association (*β*_*3*_ = -0.004, *P* = 0.452), but t-tau was significant mediator for this association (*β*_*2*_ = -0.031, *P* < 0.001). **B** Three mediation pathways were conducted between ANU-ADRI scores and MoCA: (1) ANU-ADRI → t-tau → sTREM2 → MoCA; (2) ANU-ADRI → t-tau → MoCA; (3) ANU-ADRI → sTREM2 → MoCA. The serial mediation pathway via t-tau and sTREM2 was not significant (*β*_*1*_ = -0.004, *P* = 0.324). sTREM2 was not significant mediator for this association (*β*_*3*_ = -0.005, *P* = 0.377), but t-tau was significant mediator for this association (*β*_*2*_ = -0.027, *P* = 0.002). **C** Three mediation pathways were conducted between ANU-ADRI scores and MMSE: (1) ANU-ADRI → p-tau → sTREM2 → MMSE; (2) ANU-ADRI → p-tau → MMSE; (3) ANU-ADRI → sTREM2 → MMSE. The serial mediation pathway via p-tau and sTREM2 was not significant (*β*_*1*_ = -0.002, *P* = 0.468). sTREM2 was not significant mediator for this association (*β*_*3*_ = -0.004, *P* = 0.449), but p-tau was significant mediator for this association (*β*_*2*_ = -0.023, *P* = 0.001). **D** Three mediation pathways were tested between ANU-ADRI scores and MoCA: (1) ANU-ADRI → p-tau → sTREM2 → MoCA; (2) ANU-ADRI → p-tau → MoCA; (3) ANU-ADRI → sTREM2 → MoCA. The serial mediation pathway via p-tau and sTREM2 was not significant (*β*_*1*_ = -0.002, *P* = 0.333). sTREM2 was not significant mediator for this association (*β*_*3*_ = -0.005, *P* = 0.369), but p-tau was significant mediator for this association (*β*_*2*_ = -0.018, *P* = 0.009). These three pathways are presented using green, blue, red lines. All mediation paths are adjusted for *APOE ɛ4* allele status. The β coefficients in each path and *P*-values for mediation effects were calculated by a bootstrap test with 10,000 resampling iteration. The dotted line indicates that the indirect effect is not significant (*P* ≥ 0.05), the solid line indicates that the indirect is significant (*P* < 0.05). *P < 0.05, **P < 0.01 and ***P < 0.001

CSF sTREM2, an inflammatory-related protein, showed an association with CSF p-tau level, suggesting a possible link between inflammation and tau pathology [24]. Given the mediation effects of CSF AD biomarkers observed in our mediation analyses, we thus further examined whether CSF AD biomarkers and sTREM2 mediated the effects of ANU-ADRI scores on cognition using the SEM. Therefore, three mediation pathway analyses were conducted: (1) ANU-ADRI → t-tau/p-tau → sTREM2 → MMSE/MoCA; (2) ANU-ADRI → t-tau/p-tau → MMSE/MoCA; (3) ANU-ADRI → sTREM2 → MMSE/MoCA. The model fit of the serial mediation was reported in supplementary Table 7. The results suggested that ANU-ADRI scores had a significant negative influence on MMSE/MoCA in the first model (Fig. 4 A,* β* = -0.287, *P* < 0.001; B,* β* = -0.398, *P* < 0.001; C, *β* = -0.287, *P* < 0.001; D, *β* = -0.398, *P* < 0.001). ANU-ADRI scores were positively corelated with CSF t-tau/p-tau (Fig. 4 A,* β* = 0.250, *P* < 0.001; B,* β* = 0.249, *P* < 0.001; C, *β* = 0.191, *P* < 0.001; D, *β* = 0.195, *P* < 0.001), CSF t-tau/p-tau was positively corelated with sTREM2 (Fig. 4 A,* β* = 0.374, *P* < 0.001; B,* β* = 0.393, *P* < 0.001; C, *β* = 0.333, *P* < 0.001; D, *β* = 0.346, *P* < 0.001), but sTREM2 was not significantly associated with MMSE/MoCA (Fig. 4 A,* β* = -0.031, *P* = 0.397; B,* β* = -0.037, *P* = 0.307; C, *β* = -0.027, *P* = 0.454; D, *β* = -0.037, *P* = 0.309). The indirect pathway by which ANU-ADRI scores affects MMSE/MoCA via CSF t-tau/p-tau and sTREM2 was not significant (Fig. 4 A, *β*_*1*_ = -0.003,* P* = 0.408; B, *β*_*1*_ = -0.004,* P* = 0.324; C, *β*_*1*_ = -0.002,* P* = 0.468; D, *β*_*1*_ = -0.002*, P* = 0.333). In the second mediation model, the association of ANU-ADRI scores with MMSE/MoCA was significantly mediated by t-tau/p-tau (Fig. 4 A, *β*_*2*_ = -0.031, *P* < 0.001; B, *β*_*2*_ = -0.027, *P* = 0.002 C, *β*_*2*_ = -0.023, *P* = 0.001; D, *β*_*2*_ = -0.018, *P* = 0.009). Specifically, ANU-ADRI scores were positively corelated with CSF t-tau/p-tau (Fig. 4 A,* β* = 0.243, *P* < 0.001; B,* β* = 0.240, *P* < 0.001; C, *β* = 0.191, *P* < 0.001; D, *β* = 0.194, *P* < 0.001) and CSF t-tau/p-tau was negatively corelated with MMSE/MoCA (Fig. 4 A,* β* = -0.126, *P* < 0.001; B,* β* = -0.111, *P* = 0.001; C, *β* = -0.122, *P* < 0.001; D, *β* = -0.094, *P* = 0.003). However, mediation analyses demonstrated that sTREM2 was not a significant mediator for the correlation between ANU-ADRI scores and MMSE/MoCA in the third mediation model, (Fig. 4 A, *β*_*3*_ = -0.004, *P* = 0.452; B, *β*_*3*_ = -0.005, *P* = 0.377; C, *β*_*3*_ = -0.004, *P* = 0.449; D, *β*_*3*_ = -0.005, *P* = 0.369). ANU-ADRI scores were positively corelated with sTREM2 (Fig. 4 A,* β* = 0.119, *P* = 0.002; B,* β* = 0.132, *P* = 0.001; C, *β* = 0.119, *P* = 0.002; D, *β* = 0.132, *P* = 0.001), but sTREM2 was not significantly associated with MMSE/MoCA (Fig. 4 A,* β* = -0.029, *P* = 0.414; B,* β* = -0.036, *P* = 0.331; C, *β* = -0.029, *P* = 0.410; D, *β* = -0.036, *P* = 0.327).

## Discussion

This cross-sectional study is the first to assess the relationship of ANU-ADRI scores with AD pathology in the non-demented population. Results from our study showed that ANU-ADRI scores were significantly associated with tau-related pathology and cognition and the association of ANU-ADRI scores with cognition was partially mediated by t-tau and p-tau. Besides, our SEM demonstrated that the correlation between ANU-ADRI scores with cognition was not mediated by CSF tau-related pathology and sTREM2. The evidences supported hypothesis that higher ANU-ADRI scores were related with abnormal t-tau/p-tau level and deteriorated cognition.

Our results are aligned with previous studies which demonstrated that higher ANU-ADRI scores were related with worse cognitive function in non-demented population [25]. ANU-ADRI is a reliable instrument to assess the individual AD risk and included more extensive risk and protective factors. Several meta-analyses of prospective cohorts suggested that BMI, smoking and drinking habits, as well as history of diabetes, which are part of the components of ANU-ADRI scores, were associated with dementia risk [26–29]. The cross-sectional studies have identified common risk factors for dementia and MCI in the Chinese population, including smoking, hyperlipidemia, diabetes, age, gender, and low in education [30]. The unmodifiable and modifiable risk factors were included in ANU-ADRI. Research from Venketasubramanian et al. reported that the annual incidence of dementia in Australia is higher than that in China [31]. Diet and lifestyle habits contribute differently to dementia in different ethnic groups, there were unique dietary habits in Asia to contribute to a reduced vascular risk of dementia, including drinking tea, low in sugar and calories [31]. There are several high-quality RCT studies on multi-domain interventions to prevent cognitive decline, such as FINGER, MAPT, PreDIVA [32–34]. But only FINGER provided positive results. Lifestyle interventions might be a promising option for preventing cognitive decline.

The relationships between ANU-ADRI scores and several biomarkers of AD pathology were still unexplored. After adjusting for *APOE* carrier status, our study was first to reveal the correlations between ANU-ADRI scores and CSF AD biomarkers, including t-tau, p-tau, t-tau/Aβ42, and p-tau/Aβ42. In addition, after additionally adjusting for comorbidities of coronary heart disease, stroke, as well as hypertension, our results were still significant, which was indicative of excellent robustness. Previous study has linked higher risk scores to amyloid positivity [35], but the correlation of AND-ADRI scores with CSF Aβ42 was not found in our study. The discrepancy may be due to the difference of prediction models, measurement bias of Aβ42 and sample size. As a specific markers of AD, p-tau reflects intraneuronal tangles of hyperphosphorylated tau [36, 37]. However, t-tau is a nonspecific marker of AD, and it reflects neuronal damage or intensity of degeneration [38, 39]. Consistently, several published studies have suggested a relationship of ANU-ADRI scores with AD risk [40]. Accumulating evidence demonstrated that neurodegenerative pathology might involve multiple pathways, including inflammatory, mitochondrial dysfunction, and other pathological events [41–43]. Our mediation analysis revealed that CSF t-tau and p-tau mediated the correlation of ANU-ADRI scores with MMSE or MoCA scores. Notably, the proportion of the effects mediated by CSF t-tau/p-tau was modest, but it is clear that tau pathology also partially mediated the association between ANU-ADRI scores with modifiable risk factors and cognition. Meanwhile, there might be other alternative mediators that are involved in the correlation between ANU-ADRI scores and cognition. The alternative mediators that could be considered included oxidative stress, mitochondrial dysfunction and so on. Findings from our study provided pathological basis for the relationship between ANU-ADRI scores and AD risk.

We hypothesized that ANU-ADRI scores affected cognition via tau pathology and sTREM2. However, further exploratory models demonstrated that ANU-ADRI scores were positively corelated with tau-related pathology, tau-related pathology was positively corelated with sTREM2, but sTREM2 was not significantly associated with cognition. Published studies revealed that tau pathology induced microglia activation [44, 45]. A positive relationship between CSF sTREM2 and a CSF microglial marker CCL2 provided evidence that CSF sTREM2 may represent microglial activation [46]. Previous cohort studies showed that CSF p-tau and tau pathology were associated with increased CSF sTREM2 [23, 47, 48]. Similar results were found in our study (Supplementary Table 5). The solid evidence from Taiwan revealed the correlation between plasma sTREM2 and MMSE was non-significant [49], which is consistent with our results. However, a study from ADNI database indicated that CSF sTREM2 was related to cognitive decline [50]. More researches should be carried out in different population.

### Strengths and limitations

There were two strengths in our study. We were the first to reveal the associations of ANU-ADRI scores with CSF Aβ42, t-tau, p-tau, p-tau/Aβ42, and t-tau/Aβ42, providing new insights into the mechanism underlying the association. However, the following limitations should be noted in this study. First, our study is a cross-sectional study, which implies that definitive causality could not be established. Therefore, more longitudinal cohort studies are necessary to explore these relationships in the future. Second, all results from our study are on the basis of CSF data that are less accurate than positron emission tomography (PET) imaging for measuring cerebral neurodegeneration. Third, we lacked data on three of the 15 ANU-ADRI items, including social engagement, cognitive activity, and occupational pesticide exposure, which might lead to biased results. Fourth, this study was limited to northern Chinese Han population, our findings need to be replicated in other racial/ethnic groups in the future. In addition, there were no neuropsychological assessments other than MMSE and MoCA. These cognitive screens are insensitive to subtle neuropsychological deficits.

## Conclusion

In conclusion, ANU-ADRI scores were significantly associated with CSF t-tau, p-tau, and t-tau/Aβ42 biomarkers of AD in the non-demented population. Moreover, the worse cognition associated with higher ANU-ADRI scores may be partly explained by tau pathology.

### Supplementary Information

**Supplementary Material 1.**

## Data Availability

No datasets were generated or analysed during the current study.

## Abbreviations

ANU-ADRI: Australian National University Alzheimer Disease Risk Index
AD: Alzheimer’s disease
Aβ: Amyloid-β
AgeCoDe: Study on Aging, Cognition and Dementia
ANOVA: One-way analysis of variance
*APOE** ε4*: Apolipoprotein E genotype ε4
BMI: Body mass index
CAIDE: Cardiovascular Risk Factors, Aging and Dementia
CABLE: Chinese Alzheimer’s Biomarker and LifestyLE
CN: Cognitively normal
CV: Coefficient of variation
CNS: Central nervous system
CSF: Cerebrospinal fluid
ELISA: Enzyme-linked immunosorbent assay
HAMD: Hamilton Depression Scale
MCI: Mild cognitive impairment
MMSE: Mini-Mental State Examination
MoCA: Montreal Cognitive Assessment
MLR: The multiple linear regression
P-tau: Phosphorylated tau
RCT: Randomized controlled trial
sTREM2: Soluble triggering receptor expressed on myeloid cell 2
SCD: Subjective cognitive declined
SD: Standard deviation
SEM: Structural equation model
T-tau: Total tau
TREM2: Triggering receptor expressed on myeloid cell 2

## References

[1] Long JM, Holtzman DM (2019). Alzheimer disease: an update on pathobiology and treatment strategies. Cell.

[2] Maloney B, Lahiri DK (2016). Epigenetics of dementia: understanding the disease as a transformation rather than a state. Lancet Neurol.

[3] Livingston G, Huntley J, Sommerlad A (2020). Dementia prevention, intervention, and care: 2020 report of the Lancet Commission. Lancet.

[4] Fessel J. The several ways to authentically cure Alzheimer's dementia. Ageing Res Rev 2023: 102093. 10.1016/j.arr.2023.102093 10.1016/j.arr.2023.102093 37865143

[5] Lourida I, Hannon E, Littlejohns TJ (2019). association of lifestyle and genetic risk with incidence of dementia. Jama.

[6] Rosenberg A, Ngandu T, Rusanen M (2018). Multidomain lifestyle intervention benefits a large elderly population at risk for cognitive decline and dementia regardless of baseline characteristics: The FINGER trial. Alzheimers Dement.

[7] Moll van Charante EP, Richard E, Eurelings LS (2016). Effectiveness of a 6-year multidomain vascular care intervention to prevent dementia (preDIVA): a cluster-randomised controlled trial. Lancet.

[8] Ecay-Torres M, Estanga A, Tainta M (2018). Increased CAIDE dementia risk, cognition, CSF biomarkers, and vascular burden in healthy adults. Neurology.

[9] Brett BL, Aggarwal NT, Chandran A (2023). Incorporation of concussion history as part of the LIfestyle for BRAin Health (LIBRA) modifiable factors risk score and associations with cognition in older former National Football League players. Alzheimers Dement.

[10] Jia J, Zhao T, Liu Z (2023). Association between healthy lifestyle and memory decline in older adults: 10 year, population based, prospective cohort study. BMJ.

[11] Anstey KJ, Cherbuin N, Herath PM (2013). Development of a new method for assessing global risk of Alzheimer's disease for use in population health approaches to prevention. Prev Sci.

[12] Huque MH, Kootar S, Eramudugolla R (2023). CogDrisk, ANU-ADRI, CAIDE, and LIBRA risk scores for estimating dementia risk. JAMA Netw Open.

[13] Kivimäki M, Livingston G, Singh-Manoux A (2023). Estimating dementia risk using multifactorial prediction models. JAMA Netw Open.

[14] Anstey KJ, Cherbuin N, Herath PM (2014). A self-report risk index to predict occurrence of dementia in three independent cohorts of older adults: the ANU-ADRI. PLoS One.

[15] Geethadevi GM, Peel R, Bell JS et al. Validity of three risk prediction models for dementia or cognitive impairment in Australia. Age Ageing 2022;51(12). 10.1093/ageing/afac307 10.1093/ageing/afac307 PMC9804251 36585910

[16] Stephan BCM, Pakpahan E, Siervo M (2020). Prediction of dementia risk in low-income and middle-income countries (the 10/66 Study): an independent external validation of existing models. Lancet Glob Health.

[17] De Luca SN, Chan SMH, Dobric A (2023). Cigarette smoke-induced pulmonary impairment is associated with social recognition memory impairments and alterations in microglial profiles within the suprachiasmatic nucleus of the hypothalamus. Brain Behav Immun.

[18] Yirmiya R, Rimmerman N, Reshef R (2015). Depression as a microglial disease. Trends Neurosci.

[19] Henjum K, Almdahl IS, Årskog V (2016). Cerebrospinal fluid soluble TREM2 in aging and Alzheimer's disease. Alzheimers Res Ther.

[20] Heslegrave A, Heywood W, Paterson R (2016). Increased cerebrospinal fluid soluble TREM2 concentration in Alzheimer's disease. Mol Neurodegener.

[21] Andrews SJ, Eramudugolla R, Velez JI (2017). Validating the role of the Australian National University Alzheimer's Disease Risk Index (ANU-ADRI) and a genetic risk score in progression to cognitive impairment in a population-based cohort of older adults followed for 12 years. Alzheimers Res Ther.

[22] Baron RM, Kenny DA (1986). The moderator-mediator variable distinction in social psychological research: conceptual, strategic, and statistical considerations. J Pers Soc Psychol.

[23] Ma LZ, Tan L, Bi YL (2020). Dynamic changes of CSF sTREM2 in preclinical Alzheimer's disease: the CABLE study. Mol Neurodegener.

[24] Hok AHYS, Del Campo M, Boiten WA (2023). Neuroinflammatory CSF biomarkers MIF, sTREM1, and sTREM2 show dynamic expression profiles in Alzheimer's disease. J Neuroinflammation.

[25] You J, Zhang YR, Wang HF (2022). Development of a novel dementia risk prediction model in the general population: a large, longitudinal, population-based machine-learning study. EClinicalMedicine.

[26] Anstey KJ, Cherbuin N, Budge M, Young J (2011). Body mass index in midlife and late-life as a risk factor for dementia: a meta-analysis of prospective studies. Obes Rev.

[27] Anstey KJ, Mack HA, Cherbuin N (2009). Alcohol consumption as a risk factor for dementia and cognitive decline: meta-analysis of prospective studies. Am J Geriatr Psychiatry.

[28] Xue M, Xu W, Ou YN (2019). Diabetes mellitus and risks of cognitive impairment and dementia: A systematic review and meta-analysis of 144 prospective studies. Ageing Res Rev.

[29] Zhong G, Wang Y, Zhang Y, Guo JJ, Zhao Y (2015). Smoking is associated with an increased risk of dementia: a meta-analysis of prospective cohort studies with investigation of potential effect modifiers. PLoS One.

[30] Jia L, Du Y, Chu L (2020). Prevalence, risk factors, and management of dementia and mild cognitive impairment in adults aged 60 years or older in China: a cross-sectional study. Lancet Public Health.

[31] Venketasubramanian N, Sahadevan S, Kua EH, Chen CP, Ng TP (2010). Interethnic differences in dementia epidemiology: global and Asia-Pacific perspectives. Dement Geriatr Cogn Disord.

[32] Ngandu T, Lehtisalo J, Solomon A (2015). A 2 year multidomain intervention of diet, exercise, cognitive training, and vascular risk monitoring versus control to prevent cognitive decline in at-risk elderly people (FINGER): a randomised controlled trial. Lancet.

[33] Andrieu S, Guyonnet S, Coley N (2017). Effect of long-term omega 3 polyunsaturated fatty acid supplementation with or without multidomain intervention on cognitive function in elderly adults with memory complaints (MAPT): a randomised, placebo-controlled trial. Lancet Neurol.

[34] den Brok M, Hoevenaar-Blom MP, Coley N (2022). The effect of multidomain interventions on global cognition, symptoms of depression and apathy - a pooled analysis of two randomized controlled trials. J Prev Alzheimers Dis.

[35] Pekkala T, Hall A, Ngandu T (2020). Detecting amyloid positivity in elderly with increased risk of cognitive decline. Front Aging Neurosci.

[36] Tapiola T, Alafuzoff I, Herukka SK (2009). Cerebrospinal fluid {beta}-amyloid 42 and tau proteins as biomarkers of Alzheimer-type pathologic changes in the brain. Arch Neurol.

[37] Zhao YL, Ou YN, Ma YH (2022). Association between Life's Simple 7 and cerebrospinal fluid biomarkers of Alzheimer's disease pathology in cognitively intact adults: the CABLE study. Alzheimers Res Ther.

[38] Tsitsopoulos PP, Marklund N (2013). Amyloid-β peptides and tau protein as biomarkers in cerebrospinal and interstitial fluid following traumatic brain injury: a review of experimental and clinical studies. Front Neurol.

[39] Blennow K (2004). Cerebrospinal fluid protein biomarkers for Alzheimer's disease. NeuroRx.

[40] Licher S, Yilmaz P, Leening MJG (2018). External validation of four dementia prediction models for use in the general community-dwelling population: a comparative analysis from the Rotterdam Study. Eur J Epidemiol.

[41] Franceschi C, Garagnani P, Parini P, Giuliani C, Santoro A (2018). Inflammaging: a new immune-metabolic viewpoint for age-related diseases. Nat Rev Endocrinol.

[42] Swerdlow RH (2020). The mitochondrial hypothesis: Dysfunction, bioenergetic defects, and the metabolic link to Alzheimer's disease. Int Rev Neurobiol.

[43] Atkins JL, Delgado J, Pilling LC (2019). Impact of low cardiovascular risk profiles on geriatric outcomes: evidence from 421,000 participants in two cohorts. J Gerontol A Biol Sci Med Sci.

[44] Chen X, Firulyova M, Manis M (2023). Microglia-mediated T cell infiltration drives neurodegeneration in tauopathy. Nature.

[45] Siew JJ, Chen HM, Chiu FL. et al. Galectin-3 aggravates microglial activation and tau transmission in tauopathy. J Clin Invest 2024;134(2). 10.1172/jci165523 10.1172/JCI165523 PMC10786694 37988169

[46] Deshmane SL, Kremlev S, Amini S, Sawaya BE (2009). Monocyte chemoattractant protein-1 (MCP-1): an overview. J Interferon Cytokine Res.

[47] Suárez-Calvet M, Kleinberger G, Araque Caballero M (2016). sTREM2 cerebrospinal fluid levels are a potential biomarker for microglia activity in early-stage Alzheimer's disease and associate with neuronal injury markers. EMBO Mol Med.

[48] Suárez-Calvet M, Morenas-Rodríguez E, Kleinberger G (2019). Early increase of CSF sTREM2 in Alzheimer's disease is associated with tau related-neurodegeneration but not with amyloid-β pathology. Mol Neurodegener.

[49] Tsai HH, Chen YF, Yen RF (2021). Plasma soluble TREM2 is associated with white matter lesions independent of amyloid and tau. Brain.

[50] Ewers M, Franzmeier N, Suárez-Calvet M, et al. Increased soluble TREM2 in cerebrospinal fluid is associated with reduced cognitive and clinical decline in Alzheimer's disease. Sci Transl Med 2019;11(507). 10.1126/scitranslmed.aav6221 10.1126/scitranslmed.aav6221 PMC7050285 31462511

