# Supplementary material for: ANU-ADRI scores, tau pathology, and cognition in non-demented adults: the CABLE study
Source: Alzheimers Res Ther. 2024 Mar 26;16:65. doi: 10.1186/s13195-024-01427-6 (PMC10964631; doi:10.1186/s13195-024-01427-6)
Supplement: Supplementary file 1 — Supplementary Material 1. [file 13195_2024_1427_MOESM1_ESM.docx]

**ANU-ADRI scores, tau pathology, and cognition in non-demented adults: the CABLE study**

Shan Yin, MD^1,&^, Pei-Yang Gao, MD^1,&^, Ya-Nan Ou, MD, PhD^1,&^, Yan Fu, MD, PhD^1^, Ying Liu, MD, PhD^1^, Zuo-Teng Wang, MD, PhD^1^, Bao-Lin Han, MD^1^, Prof. Lan Tan, MD, PhD^1,*^

^1^ Department of Neurology, Qingdao Municipal Hospital, Qingdao University, Qingdao 266071, China.

^&^ These authors contributed equally.

^*^ Correspondence to: Prof. Lan Tan (ORCID 0000-0002-8759-7588), Department of Neurology, Qingdao Municipal Hospital, Qingdao University, No.5 Donghai Middle Road, Qingdao, China.

E-mail address: dr.tanlan@163.com (L. Tan)

Tel: +86 21 52888163; Fax: +86 21 62483421.

**Supplementary Table 1. Associations between ANU-ADRI scores with CSF AD biomarkers and cognition**

| Variable | Aβ42 | | T-tau | | P-tau181 | | T-tau/Aβ42 | | P-tau181/Aβ42 | | MMSE | | MoCA | |
| --- | --- | --- | --- | --- | --- | --- | --- | --- | --- | --- | --- | --- | --- | --- |
|  | β | P | β | P | β | P | β | P | β | P | β | P | β | P |
| ANU-ADRI scores | 0.043 | 0.195 | 0.236 | **<0.001** | 0.183 | **<0.001** | 0.094 | **0.005** | 0.032 | 0.346 | -0.264 | **<0.001** | -0.393 | **<0.001** |

Multiple linear regression models were conducted with all models adjusted for *APOE ε4* status.

ANU-ADRI, Australian National University Alzheimer Disease Risk Index; Aβ, amyloid beta; P-tau181, phosphorylated tau181; T-tau, total tau; MMSE, Mini-Mental State Examination; MoCA, Montreal Cognitive Assessment Scale.

The statistically significant results were bolded.

**Supplementary Table 2. Sensitivity analyses of associations between ANU-ADRI scores with CSF AD biomarkers additionally adjusting for comorbidities**

| Variable | Aβ42 | | T-tau | | P-tau181 | | T-tau/Aβ42 | | P-tau181/Aβ42 | | MMSE | | MoCA | |
| --- | --- | --- | --- | --- | --- | --- | --- | --- | --- | --- | --- | --- | --- | --- |
|  | β | P | β | P | β | P | β | P | β | P | β | P | β | P |
| ANU-ADRI scores | 0.041 | 0.236 | 0.221 | **<0.001** | 0.170 | **<0**.001 | 0.088 | **0.010** | 0.027 | 0.435 | -0.266 | **<0.001** | -0.386 | **<0.001** |

Sensitivity analyses were conducted when additionally adjusting for history of coronary heart disease, hypertension and stroke.

ANU-ADRI, Australian National University Alzheimer Disease Risk Index; Aβ, amyloid beta; P-tau181, phosphorylated tau181; T-tau, total tau; MMSE, Mini-Mental State Examination; MoCA, Montreal Cognitive Assessment Scale.

The statistically significant results were bolded.

**Supplementary Table 3. Interactions analyses results**

|  | MMSE | | MoCA | |
| --- | --- | --- | --- | --- |
| ANU-ADRI**APOE ε4* | β | P | β | P |
|  | -0.09765 | 0.262 | -0.06729 | 0.425 |

ANU-ADRI, Australian National University Alzheimer Disease Risk Index; MMSE, Mini-Mental State Examination; MoCA, Montreal Cognitive Assessment Scale; *APOE ε4* apolipoprotein E genotype ε4.

**Supplementary Table 4. Associations between ANU-ADRI and sTREM2.**

| Variable | sTREM2 | |
| --- | --- | --- |
|  | β | P |
| ANU-ADRI | 0.120 | **0.001** |

Multiple linear regression models were conducted with all models adjusted for *APOE ε4* status.

ANU-ADRI, Australian National University Alzheimer Disease Risk Index; sTREM2, Soluble triggering receptor expressed on myeloid cell 2.

The statistically significant results were bolded.

| Variable | Aβ42 | | P-tau | | T-tau | | P-tau/42 | | T-tau/42 | |
| --- | --- | --- | --- | --- | --- | --- | --- | --- | --- | --- |
| sTREM2 | β | P | β | P | β | P | β | P | β | P |
|  | 0.133 | **<0.001** | 0.338 | **<0.001** | 0.380 | **<0.001** | 0.030 | 0.404 | 0.086 | **0.018** |

**Supplementary Table 5. Associations between sTREM2 and CSF AD biomarkers.**

Multiple linear regression models were conducted with all models adjusted for *APOE ε4* status.

sTREM2, Soluble triggering receptor expressed on myeloid cell 2; Aβ, amyloid beta; phosphorylated tau181; T-tau, total tau.

The statistically significant results were bolded.

**Supplementary Table 6. Assessment of cognition.**

|  | Educational years | Abnormal scores |
| --- | --- | --- |
| MMSE | >6 | ≤24 |
|  | 1-6 | ≤20 |
|  | 0 | ≤17 |
| MoCA | >6 | ≤24 |
|  | 1-6 | <19 |
|  | 0 | ≤13 |
| SCD | Do you think your memory is getting worse than it used to be? | Yes |

MMSE, Mini-Mental State Examination; MoCA, Montreal Cognitive Assessment Scale; SCD, subjective cognitive declined.

The participants with an abnormal score of MMSE or MoCA were considered to be the MCI group. According to the SCD-1 suggestion, “Do you think your memory is getting worse than it used to be?” was a dichotomous question that can not be accounted for by other diseases or substance addiction, used to assess whether participants belonged to the SCD group. Participants with neither SCD or MCI were defined as CN group.

**Supplementary Table 7. The fitting index of sTREM2 and t-tau/p-tau intermediation models**

|  | GFI | CFI | SRMR | RMSEA |
| --- | --- | --- | --- | --- |
| ANU-ADRI->t-tau->sTREM2->MMSE | 0.991 | 0.969 | 0.025 | 0.071 |
| ANU-ADRI->t-tau->sTREM2->MoCA | 0.993 | 0.98 | 0.023 | 0.064 |
| ANU-ADRI->P-tau->sTREM2->MMSE | 0.987 | 0.939 | 0.032 | 0.09 |
| ANU-ADRI->P-tau->sTREM2->MoCA | 0.991 | 0.969 | 0.027 | 0.073 |

ANU-ADRI, Australian National University Alzheimer Disease Risk Index; sTREM2, Soluble triggering receptor expressed on myeloid cell 2; P-tau, phosphorylated tau; T-tau, total tau; MMSE, Mini-Mental State Examination; MoCA, Montreal Cognitive Assessment Scale; GFI, goodness-of-fit index (> 0.9); CFI, Comparative Fit Index (> 0.9); SRMR, Standardized Root Mean squared Residual (< 0.05); RMSEA, Root Mean Squared Error of Approximation (< 0.05).

**Supplementary Figure 1**


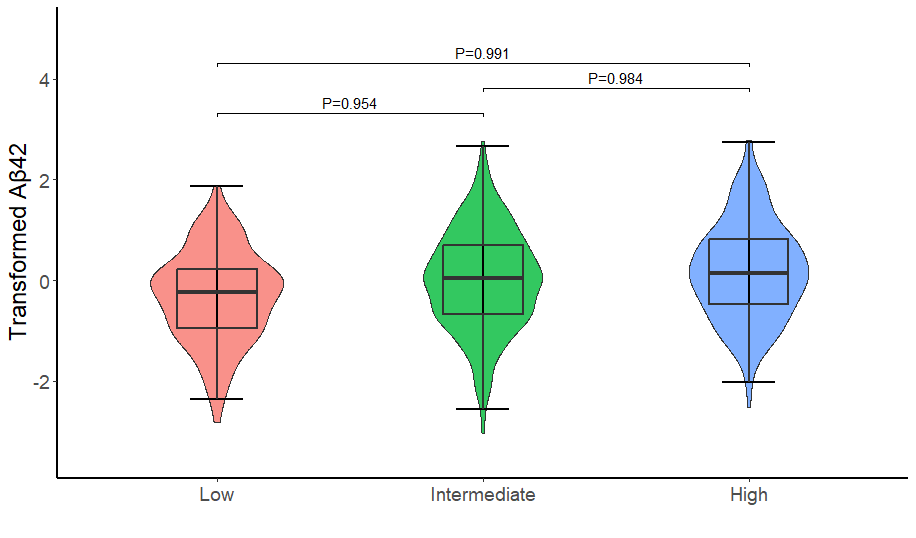


Differences in CSF Aβ42 were examined by the analysis of variance. Aβ, amyloid beta.

**Supplementary Figure 2**

**
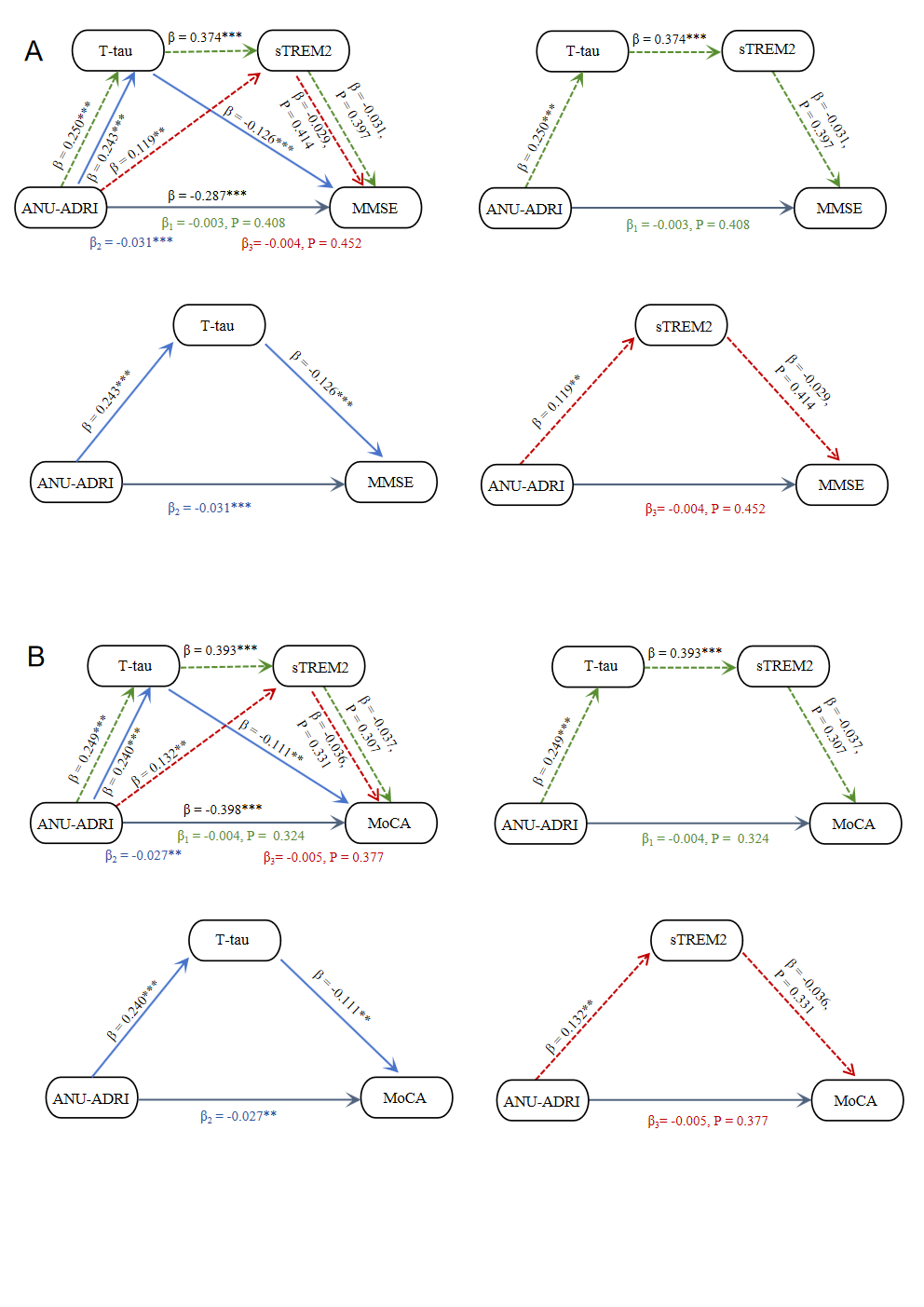
**

**
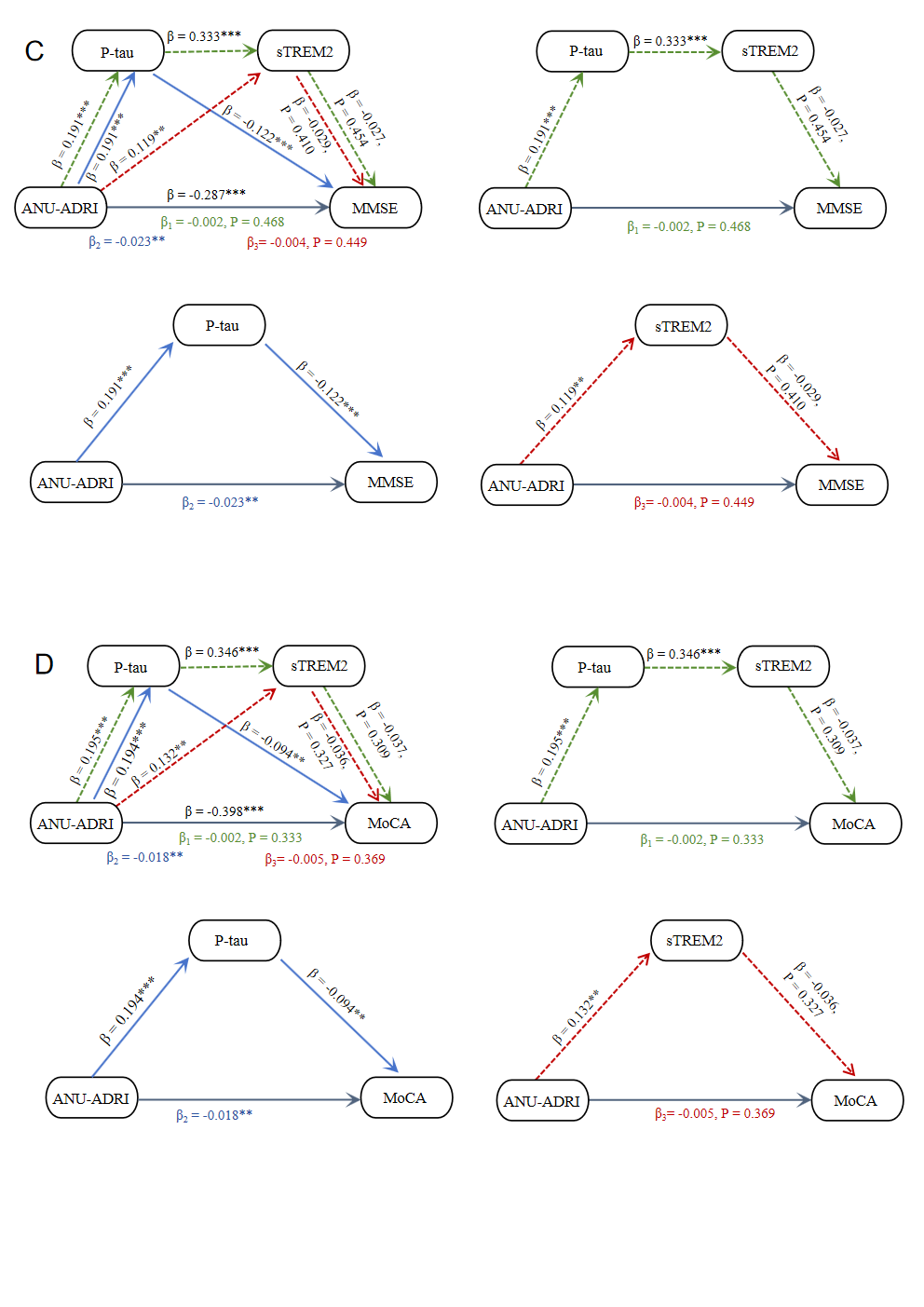
**

All estimates for each model are presented in two ways. **A.** Three mediation pathways were conducted between ANU-ADRI scores and MMSE: (1) ANU-ADRI → t-tau → sTREM2 → MMSE; (2) ANU-ADRI → sTREM2 → MMSE; (3) ANU-ADRI → t-tau → MMSE. The serial mediation pathway via t-tau and sTREM2 was not significant (*β_1_* = -0.003, *P* = 0.408). sTREM2 was not significant mediator for this association (*β_3_* = -0.004, *P* = 0.452), but t-tau was significant mediator for this association (*β_2_* = -0.031, *P* < 0.001). **B**. Three mediation pathways were conducted between ANU-ADRI scores and MoCA: (1) ANU-ADRI → t-tau → sTREM2 → MoCA; (2) ANU-ADRI → sTREM2 → MoCA; (3) ANU-ADRI → t-tau → MoCA. The serial mediation pathway via t-tau and sTREM2 was not significant (*β_1_*= -0.004, *P* = 0.324). sTREM2 was not significant mediator for this association (*β_3_* = -0.005, *P* = 0.377), but t-tau was significant mediator for this association (*β_2_* = -0.027, *P* = 0.002). **C.** Three mediation pathways were conducted between ANU-ADRI scores and MMSE: (1) ANU-ADRI → sTREM2 → p-tau → MMSE; (2) ANU-ADRI → sTREM2 → MMSE; (3) ANU-ADRI → p-tau → MMSE. The serial mediation pathway via t-tau and sTREM2 was not significant (*β_1_*= -0.002, *P* = 0.468). sTREM2 was not significant mediator for this association (*β_3_* = -0.004, *P* = 0.449), but t-tau was significant mediator for this association (*β_2_* = -0.023, *P* = 0.001). **D.** Three mediation pathways were tested between ANU-ADRI scores and MoCA: (1) ANU-ADRI → sTREM2 →p-tau →MoCA; (2) ANU-ADRI → sTREM2 → MoCA; (3) ANU-ADRI →p-tau → MoCA. The serial mediation pathway via t-tau and sTREM2 was not significant (*β_1_*= -0.002, *P* = 0.333). sTREM2 was not significant mediator for this association (*β_3_* = -0.005, *p* = 0.369), but t-tau was significant mediator for this association (*β_2_* = -0.018, *P* = 0.009). These three pathways are presented using green, blue, red lines. All mediation paths are adjusted for *APOE ɛ4* allele statuses. The β coefficients in each path and P-values for mediation effects were calculated by a bootstrap test with 10,000 resampling iteration. The dotted line indicates that the indirect effect is not significant (*P* ≥ 0.05), the solid line indicates that the indirect is significant (*P* < 0.05). **P*<0.05, ***P*<0.01 and ****P*<0.001
